# Unlocking the Potential of Biomarkers for Immune Checkpoint Inhibitors in Cancer Therapy

**DOI:** 10.3390/cancers15184503

**Published:** 2023-09-10

**Authors:** Giada Dal Collo, Paul Takam Kamga

**Affiliations:** 1 Department of Immunology, Erasmus University Medical Center, 3015 GD Rotterdam, The Netherlands; giada.dalcollo@gmail.com; 2 EA4340 BECCOH, Université Paris-Saclay, UVSQ92100 Boulogne-Billancourt, France

Immune checkpoint inhibitors (ICIs) are pharmaceutical agents capable of disrupting immune checkpoint signaling, leading to T-cell activation and a robust anti-tumor response [1]. The first anti-cancer ICI was aimed at targeting Cytotoxic T-Lymphocyte Associated Protein 4 (CTLA4) (Ipilimumab). It showed promising outcomes in pre-treated patients with melanoma, including anticancer response rates and improved overall survival (OS) [1]. Subsequent advancements yielded a range of ICIs focusing on Programmed cell death protein-1/Programmed death-ligand 1 (PD1/PDL1) (i.e., Pembrolizumab, Atezolizumab, Durvalumab, and Nivolumab), which have been utilized as primary treatments for refractory melanoma, advanced and metastatic NSCLC, and other malignancies encompassing solid tumors and hematological malignancies. All these ICIs have demonstrated clinical advantages in terms of objective response rate (ORR) and survival, resulting in first-line therapeutic options administered alone or in conjunction with complementary strategies such as chemotherapy or radiotherapy [2,3,4]. Nonetheless, not all patients respond equally to these treatments, prompting the need for predictive biomarkers to optimize treatment selection and elevate patient outcomes. In addition, ICIs are associated with immune-related adverse events (IRAEs). These events not only induce patient discomfort but can also compel the temporary or permanent halt of immunotherapy [5]. Because many of these IRAEs are intricately tied to the mechanisms underlying ICIs, the pursuit of predictive biomarkers for IRAEs holds immense significance and often intersects with biomarkers used to evaluate ICIs’ effectiveness [6,7,8]. Notably, evidence from studies suggests that IRAEs correlate with substantial enhancements in ORR and patient survival, underscoring their predictive value [9].

PDL-1 expression, as assessed through immunohistochemistry (IHC), was the first marker to gain approval and practical application in conjunction with ICIs. Patients with high PDL-1 expression (PDL-1 > 50%) tend to exhibit a more favorable response to the treatment. However, intriguingly, certain patients with lower PDL-1 expression (PDL-1 < 10%; PDL-1 < 5%; PDL-1 < 1%) have demonstrated enhanced ORR and prolonged survival, while some with elevated PDL-1 expression exhibited resistance to therapy [10,11,12]. Furthermore, it is noteworthy that the predictive value of PDL-1 expression does not remain consistent across all types of ICIs and cancer varieties [13]. This disparity arises, in part, from the spatiotemporal heterogeneity of the tumor, the variations in the methods utilized to assess PDL-1 expression, including techniques for IHC, the types of cells analyzed (cancer cells, immune cells, or both), and the specific antibody type employed [14]. As a result, there is a pressing need to standardize and refine the methods for analyzing PDL-1 expression. Circulating soluble PDL-1 (sPDL-1) has displayed encouraging potential. Despite certain contrasting findings, systematic reviews and meta-analyses provided evidence for a positive correlation between high levels of sPDL-1 and worse survival in individuals undergoing ICI treatment for solid cancer, including NSCLC and gastric cancer [15,16,17]. As previously mentioned, it is evident that PDL-1 expression alone is insufficient for patient stratification. Hence, in addition to PD-L1 expression, several other biomarkers are currently in the process of being developed.

Tumor mutational burden (TMB) quantifies the number of mutations harbored by tumor cells within a specific neoplasm. This mutational burden is closely associated with antigen processing and load in MHC-I, consequently impacting anti-tumoral immunity. For this instance, the study of Robert M. Samstein et al. demonstrated a positive correlation between high TMB and efficacy of ICIs across diverse cancer types and for different types of ICIs, as revealed by better ORR and longer survival in a robust cohort of 1662 cancer patients treated with different ICIs (Atezolizumab, Avelumab, Durvalumab, Ipilimumab, Nivolumab, Pembrolizumab, or Tremelimumab) [18]. This conclusion was further confirmed in several studies, as demonstrated by the systematic analysis of Kim et al., who examined 26 studies focused on immune checkpoint inhibitors (ICIs) in cancer. All these studies converge to show a correlation between high TMB, better response, longer overall survival (OS), and progression-free survival (PFS). This collective evidence supports the idea that TMB could serve as an effective biomarker associated with the use of ICIs [19,20]. 

The genetic variation in the DNA mismatch repair (MMR) pathway is one source of a high TMB load, which can lead to genome instability and microsatellite instability (MSI). For instance, as revealed by the Phase II CheckMate 142 Study, the application of ICIs in metastatic colorectal cancer (mCRC) demonstrated that defects in MMR and/or MSI (dMMR/MSI-H) are predictive indicators for the effectiveness of Pembrolizumab, Nivolumab, and Nivolumab in combination with low-dose Ipilimumab [21,22]. Notably, this correlation has also been observed across various other cancer types, including lung cancer, melanoma, renal cell carcinoma, and many others [23,24]. Additionally, as suggested by Schrock et al. and validated by emerging data, the utilization of TMB in conjunction with dMMR/MSI-H enhances the predictive value for ICIs compared to using dMMR/MSI-H alone [24]. Nevertheless, the pragmatic implementation of TMB in the clinical care of patients poses challenges. For instance, the use of genome sequencing or advanced next-generation sequencing tools to assess TMB load, though informative, can introduce complexity and prove burdensome when integrating them into everyday clinical routines [25]. Although the use of next-generation sequencing for targeted gene panels has somewhat alleviated this burden, the lack of a well-defined consensus on the cutoff ratio across various studies underscores the need for harmonization efforts [26]. 

In the pursuit of identifying alternative biomarkers, researchers have directed their efforts towards analyzing intermolecular interactions within tumor cells [27]. This exploration has extended to encompass the evaluation of various molecules, i.e., signaling molecules, cytokines/chemokines, and different cell types present in the tumor microenvironment, including tumor-associated fibroblasts (CAF), tumor-associated macrophages (TAM), mesenchymal stroma cells, and infiltrating immune cells. Additionally, it encompasses the examination of circulating and systemic markers within the host [4,28,29]. These encompass immune gene signatures [30], tumor-infiltrating lymphocytes (TILs), diverse T cell populations (such as CD8+, regulatory T cells, and T helper cells) [24,25], myeloid-derived suppressor cells (MDSCs) [31], and even the composition of the gut microbiome [32]. Thanks to advanced omics techniques, advancements in computational tools, the application of artificial intelligence, and a systems biology approach, emerging studies combine multiple markers to define expression patterns or nomograms that may accurately predict the outcomes of ICIs [30,33,34]. However, while the clinical feasibility of such an approach, as well as the utilization of single markers, necessitate further investigation, the validation and standardization of these methodologies across different cancer types and treatment contexts remain crucial challenges. 

A majority of the biomarkers currently under investigation encompass cellular factors or molecules intricately engaged in immune evasion. These components operate within a coordinated framework, either fostering an environment favoring anti-inflammatory processes or contributing to the creation of a suppressive tumor niche that hampers anticancer immunity, ultimately culminating in resistance to ICIs [35,36]. The current challenges reside in effectively implementing experimental models that replicate interactions among immune, stromal, and cancer cells. For instance, the classic two-dimensional (2D) coculture models often entail cultivating immune effector cells (T, B, and NK cells) on monolayers of cancer or stromal cells, encompassing various components such as MSCs, fibroblasts, and macrophages [37]. This model is frequently complemented by animal models or three-dimensional (3D) culture systems and organs-on-chips, offering the advantage of emulating the intricate cellular dynamics and mechanical complexity observed in patients [38,39,40]. By integrating these preclinical models with advanced high-resolution imaging and real-time monitoring, a complete comprehension of the dynamic interactions can be achieved, not only deepening our understanding of the mechanisms underlying resistance to ICIs, but also highlighting potential drug targets that could enhance the efficacy of ICIs [40,41].

In conclusion, the collaborative efforts of researchers and clinicians in deciphering the intricacies of immune responses offer a pathway to personalized treatment strategies driven by biomarkers. This holds the potential to enhance the effectiveness of cancer therapy and ultimately lead to better patient outcomes. A holistic endeavor towards validating, standardizing, and incorporating biomarkers will pave the road towards achieving precision medicine within the realm of immune checkpoint inhibitors and cancer treatment.
